# A dataset of organic pollutants identified and quantified in recycled polyethylene pellets

**DOI:** 10.1016/j.dib.2023.109740

**Published:** 2023-10-30

**Authors:** Eric Carmona, Elisa Rojo-Nieto, Christoph D. Rummel, Martin Krauss, Kristian Syberg, Tiffany M Ramos, Sara Brosche, Thomas Backhaus, Bethanie Carney Almroth

**Affiliations:** a Department of Biological and Environmental Sciences, University of Gothenburg, 405 30 Gothenburg, Sweden; b Department of Effect-Directed Analysis. Helmholtz-Centre for Environmental Research – UFZ. Permoserstraße 15, 04318 Leipzig, Germany; c Department of Ecological Chemistry. Helmholtz-Centre for Environmental Research – UFZ. Permoserstraße 15, 04318 Leipzig, Germany; d Department of Science and Environment, Roskilde University, Universitetsvej 1, 4000, Roskilde, Denmark; e IPEN, The International Pollutants Elimination Network, Första Långgatan 18, 413 28, Gothenburg, Sweden; f RWTH Aachen University, Worringerweg 1, 52074 Aachen, Germany

**Keywords:** Recycled plastics pellets, Contaminants of emerging concern, High-density polyethylene, Non-intentionally added substances, Plastic additives

## Abstract

Plastics are produced with a staggering array of chemical compounds, with many being known to possess hazardous properties, and others lacking comprehensive hazard data. Furthermore, non-intentionally added substances can contaminate plastics at various stages of their lifecycle, resulting in recycled materials containing an unknown number of chemical compounds at unknown concentrations. While some national and regional regulations exist for permissible concentrations of hazardous chemicals in specific plastic products, less than 1 % of plastics chemicals are subject to international regulation [1]. There are currently no policies mandating transparent reporting of chemicals throughout the plastics value chain or comprehensive monitoring of chemicals in recycled materials.

The dataset presented here provides the chemical analysis of 28 samples of recycled High-Density Polyethylene (HDPE) pellets obtained from various regions of the Global South, along with a reference sample of virgin HDPE. The analysis comprises both Target and Non-Targeted Screening approaches, employing Liquid Chromatography-High-Resolution Mass Spectrometry (LC-HRMS) and Gas Chromatography-High-Resolution Mass Spectrometry (GC-HRMS). In total, 491 organic compounds were detected and quantified, with an additional 170 compounds tentatively annotated. These compounds span various classes, including pesticides, pharmaceuticals, industrial chemicals, plastic additives.

The results highlight the prevalence of certain chemicals, such as N-ethyl-o-Toluesulfonamide, commonly used in HDPE processing, found in high concentrations. The paper provides a dataset advancing knowledge of the complex chemical composition associated with recycled plastics.

Specifications Table

**Table:** 

Subject	Environmental science - Pollution
Specific subject area	Organic chemicals in High Density Polyethylene recycled plastic pellets.
Data format	Raw, Analysed, Filtered
Type of data	Tables
Data collection	Recycled plastics pellets were collected from facilities in the Global South. Polymers were confirmed using attenuated total reflection Fourier transform infrared (ATR-FTIR) spectroscopy. Organic chemicals were extracted using an Ultrasound Assisted Extraction, analysed by High Performance Liquid Chromatograph and Gas Chromatograph devices in tandem with Q-Exactive Mass Spectrometers. X The data was analysed with the software Thermo TraceFinder and a workflow using MZmine and the R-Script MZquant.
Data source location	*The 28 analysed recycled PE plastic pellets are from recycle treatment plants from the following countries:* • *Argentina* • *Cameroon* • *India* • *Indonesia* • *Malaysia* • *Mauritius* • *Nepal* • *Nigeria* • *Serbia* • *Taiwan* • *Tanzania* • *Thailand* • *Togo* *The samples were purchased by different NGOs (Non-Governmental Organisations) operating locally, but the specific sites of the recycling facilities and names of companies are not revealed (see Limitations).*
Data accessibility	Repository name: Zenodo Data identification number: 10.5281/zenodo.8367104 Direct URL to data: https://doi.org/10.5281/zenodo.8367104

## 1 Value of the Data

• 13 000 chemicals are currently known to be used in the production of plastics materials and products [2]. Several of these chemicals have hazardous properties while thousands of the chemicals lack data, even basic toxicological data. Non-intentionally added substances may further contaminate plastics during production, use, waste and recycling stages of the plastics value chain, resulting in recycled materials that contain an unknown number of chemical substances in unknown concentrations.
• While national and regional policies regulate permissible concentration of hazardous chemicals in specific plastics products, only 1% of plastics chemicals are regulated internationally. There are few policies requiring transparent reporting of chemicals in plastics throughout their value chain. Nor is thorough monitoring of chemicals in recycled materials legally mandated and implemented.
• Data on the types and concentrations of chemicals in recycled plastics, in particular the non-intentionally added substances, are critical for the quality assessment of recycled plastics and can inform regulations that specify in which recycled plastics can be used. The data provided will therefore be useful to actors in numerous sectors throughout the plastics value chain, including producers and manufacturers, regulators and policy makers, workers in production and waste management, including recycling, consumers, as well as the scientific community.
• Researchers in several fields of study will also find this information useful. This includes natural sciences, e.g., chemical engineering and material sciences, (eco)toxicology, human health, epidemiology as well as social sciences addressing policy, environmental behaviour, anthropology, economists and legal scholars addressing chemicals and plastics regulations.

## 2 Data Description

This dataset offers the chemical analysis of 28 different recycled plastic pellets produced and purchased in the Global South, in countries in Africa, South-America, Asia as well as one country in Europe. When purchasing samples from recycling facilities, we requested high density polyethylene (HDPE) pellets. Results of the FT-IR analyses are found in the Zenodo repository in **Tables 004-263**, and all samples were confirmed to consist of HDPE. Furthermore, a pellet produced from virgin HDPE was analysed in order to compare with the recycled samples and used as sample blank. A target analysis with 625 organic compounds for the LC-HRMS and 200 for the GC-HRMS was performed. 491 compounds were detected and quantified (**Table 2 in Zenodo - Results Target**). A Non-Targeted Screening (NTS) analysis was also performed and around 170 compounds were tentatively annotated in different orders of identification [3] (**Table 3 in Zenodo - Non-Target Screening**).

The majority of the detected pollutants in the Target Screening are pesticides, followed by pharmaceuticals and industrial compounds (Table 1). These chemical classes represent the majority of entries in the target database.

**Table 1:** Overview of the numbers of different chemicals detected in target analysis, indicated by class, detected at least in one sample.

Class of chemical	Number
Pesticides and biocides	162
Pharmaceuticals	89
Industrial chemicals	65
Plastic additives	45
Polycyclic aromatic hydrocarbons (PAHs)	21
Food ingredient	12
Polychlorinated biphenyls (PCBs)	12
Surfactants	10
Fragrances	8
UV filters	6
Dye	4
Stimulants	4
Corrosion inhibitor	3
Polybrominated diphenyl ethers (PBDEs)	2
Repellents	2
Human metabolites	2
Polychlorinated naphthalene (PCNs)	1

The compound found in the highest concentration was N-Ethyl-o-toluenesulfonamide, a common plasticizer which was found in all the samples, with a maximum concentration of 24020µg/L of extract. The chemical with the second highest concentration is the rubber additive N,N-Dimethyl-p-phenylenediamine, with a maximum concentration of 77463 ng/L of the extract.

## 3 Experimental Design, Materials and Methods

### 3.1 Samples

A total of 28 plastic pellet samples were collected from small scale plastics recycling facilities from countries in South America, Asia, Africa, and Europe. We are reporting the names of the countries but not providing any additional information on site locations due to security reasons (see section on limitations). Additionally, virgin HDPE pellets were purchased from Sigma-Aldrich to serve as a reference material.

### 3.2 Polymer composition

Fourier transform infrared (ATR-FTIR) spectroscopy analyses were conducted (FT-IR Spectrometer, Frontier, PerkinElmer) to validate polymer composition of pellet samples. Each sample contained 10 replicate pellets which were each assessed at a single point with the FTIR, results for each sample can be found in Tables 004-263. The polymer composition of each sample was based on a ≥ 90% match with reference spectra, in addition to manual assessment of compliance for peaks within 1400–4000 cm^-1^ range of the spectra using Spectrograph (version 1.2) [2,3].

### 3.3 Chemicals and reagents

For the mobile phases at the LC, LC–MS grade methanol, formic acid and ammonium formate were purchased from Honeywell and LC–MS grade water from Thermo-Fisher. LC–MS grade ethyl acetate and 7 N ammonia in methanol were obtained from Sigma-Aldrich. All samples were analysed for 610 chemicals (including 150 retrospectively) with a consistent target screening approach using liquid chromatography high-resolution mass spectrometry (LC-HRMS). The list of target compounds is based on organic compounds that are usually monitored in water compartments. The standards were purchased from different suppliers as detailed in Nanusha 2020 [4].

A matrix matched calibration in ethyl acetate (EtAc) was used for the quantification of the GC analysis. Gas chromatography grade ethyl acetate was purchased from Merck (Darmstadt, Germany). Analytical standards were purchased from Dr. Ehrenstorfer (Augsburg, Germany) and Sigma-Aldrich (Germany). Internal Standards (IS) were purchased from Wellington Laboratories (Guelph, ON, Canada) and Campro Scientist (Berlin, Canada).

### 3.4 Sample extraction and analysis

The HDPE pellets were extracted by Ultrasound Assisted Extraction (UAE), using three different organic solvents: Methanol (MeOH), a solution of Acetonitrile (ACN):MeOH (2:1) and Hexane (Hx). 500 mg of sample were extracted 6 times, two for each combination of the following solvents: first, the samples were extracted, twice with 4 mL MeOH over 2 × 15 min, allowing to cool down between sonication using an ice bath. The same procedure was carried out afterwards using 4 mL of ACN:MeOH twice, during 2 × 15 min, with intermedia cooling down. A third round of extractions was carried out on the sample, with Hx (4 mL, twice, 2 × 15 min sonication, with cooling down between sonication). The resulting solution compiling all the aliquots was evaporated until dryness and reconstituted with a) MeOH:H_2_O (70:30) for LC-HRMS analysis and b) ethyl acetate for GC-HRMS.

Liquid-chromatographic separation was performed on a Kinetex C18 EVO column (50 × 2.1 mm, 2.6 µm in particle-size) from Phenomenex (Torrance, CA, USA) equipped with a pre-column (C18 EVO 5 × 2.1 mm) and an inline filter as described by Nanusha et al. [5]. The mobile phase consisted of 0.1% of formic acid (eluent A) and methanol containing 0.1% of formic acid (eluent B), which was used at a flow rate of 300 µL/min. After 1 min of elution with 5% of eluent B, the fraction of eluent B was linearly increased to 100% within 12 min, after which the mobile phase was kept at 100% of eluent B for 11 min. Subsequently, the column was rinsed with a mixture of isopropanol + acetone 50:50/eluent B/eluent A (85%/10%/5%) to remove hydrophobic-matrix constituents from the column. Finally, the column was re-equilibrated to initial conditions for 5.7 min. An injection volume of 5 µL was used, and the column was operated at 40 °C. The heated ESI source and the transfer capillary were both operated at 300 °C with a spray voltage of 3.8 kV, a sheath gas-flow rate of 45 a.u., and an auxiliary gas-flow rate of 1 a.u. The full-scan MS1 was recorded in the m/z range of 100–1500 with a nominal resolving power of 140,000 (referenced to m/z 200). For compound confirmation, data-dependent MS/MS acquisition was performed at a resolving power of 70,000 in additional runs. The MS was calibrated externally every 2 days using the calibration mixtures of the vendor. The mass accuracy was always within 5 ppm for all analyses. All MS and MS/MS analyses were performed in ESI positive (ESIpos) and ESI negative (ESIneg) modes.

In the case of the semi-volatile hydrophobic analytes, the separation was achieved with a GC system consisting of a TriPlus RSH autosampler with a Trace 1310 GC coupled with a Thermal Desorption Unit (TDU-2) and a Cooled Injection System (CIS, both from Gerstel, Mülheim, Germany). The method has been described earlier by Muz et al. [6] and Reiter et al. [7] The injections were made in splitless mode with an injection volume of 2 µL. Helium was used as carrier gas at a constant flow of 1.2 mL/min. The thermal desorption in the TDU was carried out with the heating program from 30 °C to 300 °C at a heating rate of 300 °C/min (holding 5 min). The transfer temperature of the TDU on the top of the CIS was set at 320 °C. After refocusing in the glass liner with deactivated glass wool (CIS-4 TDU, Gerstel, Mülheim, Germany) at 10 °C for 0.2 min, the analytes were desorbed with a temperature of up to 300 °C at a fast rate of 12 °C/s and a final holding time of 10 min and injected in a splitless mode with a time of 2 min. The chromatographic separation was based on the following temperature program: 60 °C (1 min), up to 150 °C at 30 °C /min, up to 186 °C at 6 °C/min rate, up to 300 °C with a rate of 4 °C/min (holding 11.5 min). The GC was coupled with a QExactive instrument (Thermo Fisher Scientific, Germany) via a transfer line kept at 280 °C. The ion source temperature was set at 250 °C. Mass spectrometric analysis was performed using electron ionisation (EI) at 70 eV in positive polarity, in full-scan mode with a scan range of 70–810 m/z and a resolution of 60,000 (FWHM at m/z 200). The internal calibration and tuning of the instrument were established using Perfluorotributylamine (PFTBA) as a mass calibrant.

### 3.5 Data analysis

Three data analysis methods were applied to the data (see Table 2 in the Zenodo repository). ProteoWizard (version 3.0.19324-f948194c2) was used to convert the LC–HRMS raw data into mzML format (centroid mode) [8] for the Target Analysis. Subsequently, peak detection, sample alignment, and target-compound annotation were performed using MZmine (V 2.38) [9], as detailed in [10]. An R package (MZquant, version 0.7.8, Schulze, 2021a) [11] was used to perform blank correction, calibration, and quantification. Blank-peak elimination and blank-intensity thresholds were calculated according to the procedures conducted in Machate et al. [12]. Lastly, a series of calibration standards ranging from 0.5 to 5000 ng/L were used. The calibration standards were treated the same way as the plastic samples, including the same concentration of Internal Standards. The target compounds were quantified using the internal standards with the nearest retention time.

TraceFinder 4.1 from Thermo Scientific was used to analyse the data that was acquired with the Gas Chromatography Mass Spectrometry, integrating the peak areas (in Table 2 of the Zenodo repository, results target, GC-TF). Calibration standards from 1 ng/mL to 500 ng/mL were used to calculate the concentrations.

Additionally, a retrospective analysis was performed (MZQ-R19) in order to identify and quantify around 200 compounds from the LC-HRMS analysis. An R-script using the packages *tidyverse* and *xcms* to get the Retention Times (RT) directly from the mzML files. After correcting the RT, the same workflow with MZmine and MZquant was implemented, as detailed above.

Finally, the software Compound Discoverer 3.3 from Thermo Scientific was used to perform the Non-Targeted Screening data analysis, with a workflow to identify chemical additives and environmental pollutants using mzCloud and ChemSpider as annotation libraries.

## 4 Limitations

Samples were purchased from recycling facilities, where we only have cursory knowledge of the plastic waste streams used as feedstock for the production of the analysed pellets. The locations of the recycling facilities and the names of people involved in purchasing the samples are not made public, to ensure the privacy of the individuals involved, as well as recycling facilities, and to avoid any safety concerns.

## Ethics Statement

The authors have read and followed the ethical requirements for publication in Data in Brief and confirm that the current work does not involve human subjects, animal experiments, or any data collected from social media platforms.

## CRediT authorship contribution statement

**Eric Carmona:** Conceptualization, Data curation, Formal analysis, Investigation, Methodology, Software, Visualization, Writing – original draft, Writing – review & editing. **Elisa Rojo-Nieto:** Methodology, Writing – review & editing. **Christoph D. Rummel:** Methodology. **Martin Krauss:** Methodology, Validation, Writing – review & editing. **Kristian Syberg:** Formal analysis, Data curation, Writing – review & editing. **Tiffany M Ramos:** Formal analysis, Data curation, Writing – review & editing. **Sara Brosche:** Conceptualization, Resources. **Thomas Backhaus:** Conceptualization, Writing – review & editing. **Bethanie Carney Almroth:** Conceptualization, Project administration, Funding acquisition, Supervision, Writing – review & editing.

## Data Availability

A dataset of organic pollutants identified and quantified in recycled polyethylene pellets (Original data) (Zenodo)
